# Childhood life events of women enrolled in the Avon Longitudinal Study of Parents & Children (ALSPAC)

**DOI:** 10.12688/wellcomeopenres.19459.1

**Published:** 2023-07-10

**Authors:** Steven Gregory, Yasmin Iles-Caven, Kate Northstone, Jean Golding

**Affiliations:** 1 Population Health Sciences, Bristol Medical School, University of Bristol, Bristol, England, BS8 2BN, UK

**Keywords:** ALSPAC, ACEs, trauma, parent, childhood, behaviour, stress, longitudinal cohort

## Abstract

At the time of planning ALSPAC there was accumulating evidence that abuse and other childhood traumas were related to psychiatric problems later in life. In addition, the age at which such trauma occurred was likely to be important in influencing its long-term impact. Detailed data was therefore collected from enrolled women on traumatic events occurring during their own childhoods, along with their age at the time.

The questionnaire entitled ‘About Yourself’ was sent out to expectant women who had enrolled in the study, which included a page in the form of a grid (an events diary) with one row per year of childhood and columns for recording where she was living at the time, who was looking after her, and any traumatic events that occurred. These free-text responses were then coded, and any events were assigned a score indicating the level of trauma the event was likely to have caused on a scale of 1 (highly traumatic) to 6 (least traumatic).  This paper describes the variety of text data collected and how it was coded.

The ALSPAC study has a great deal of follow-up data collected on the original respondents, as well as on their parents and grandparents, partners, offspring and their grandchildren, providing huge potential for analyses on the antecedents and outcomes of adverse childhood events across multiple generations.

## Introduction

There is accumulating evidence that psychological abuse and other traumas occurring during childhood are related to psychiatric problems in the adult, and that the age at which such traumas occurred is likely to be important in influencing their long-term impact (see Rutter & Maughan, 1997 for a discussion of the need for further research). These Adverse Childhood Events (ACEs) may include childhood physical and sexual abuse, traumatic accidents, being a witness to distressing events (such as those resulting in a death, causing serious injury or posing a threat to own physical safety, serious illness and bereavement, etc.) (e.g., Nelson & Wampler, 2007). ACEs have been shown to increase the incidence of depression, anxiety and post-traumatic stress disorder in adulthood (e.g., Singer *et al.*, 1995).

As ACEs influence mental health, this can have a knock-on effect on the next generation. For example, previous research using data from the Avon Longitudinal Study of Parents and Children (ALSPAC) has found an association between maternal ACEs and harsh parenting and bullying, and increased depressive symptoms, anxiety and low self-esteem in their children ( Fisher *et al.* 2013).

**Figure 1.**
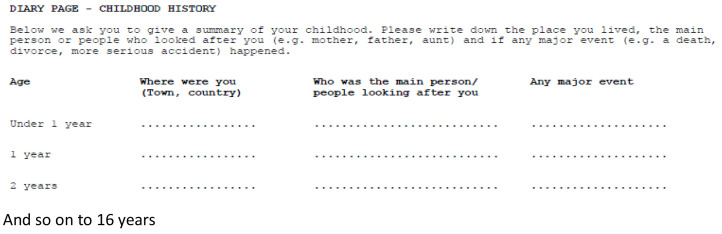
The mothers’ childhood events diary.

During the early stages of planning the data collection in ALSPAC, it was suggested by Professor Sir Michael Rutter (a member of the ALSPAC Scientific Advisory Committee and a collaborator) that, not only would it be valuable to collect detailed data on the women during her own childhood, but it would also be particularly useful to be able to identify the age at which any traumatic event had occurred. He suggested that the data should be collected in the form of an events diary, with a line for each year of her childhood from less than 1 year to 16 years of age ( Figure 1).

This paper describes the text data collected in this diary, which allowed the women to record any information which they felt to be relevant. ALSPAC also collected data on traumatic events in the womens’ childhoods by asking them directly about specific events (with tick box answers). These included: death of a parent, being seriously ill, physical or mental cruelty by a parent, sexual abuse, among others. These variables are not within the scope of this paper, and have previously been described ( Ellis *et al.*, 2020). Adverse events in the childhoods of the offspring cohort have also been described elsewhere ( Houtepen *et al.*, 2018). Additionally, the parent cohort were asked to describe traumatic events in the lives of their own parents and grandparents and an insight into the variety of responses has been published ( Birmingham *et al.*, 2021).

## Methods

### Participants

***The Avon Longitudinal Study of Parents and Children (ALSPAC)*.** Pregnant women resident in Avon in the south-west of England with expected dates of delivery between 1st April 1991 and 31st December 1992 were invited to take part in ALSPAC. The initial number of enrolled pregnancies was 14,541. Of these, there was a total of 14,676 fetuses, resulting in 14,062 live births and 13,988 children who were alive at 1 year of age. The phases of enrolment are described in more detail in the cohort profile paper and its updates ( Boyd *et al.*, 2013; Fraser *et al.*, 2013.). Please note that the study website contains details of all the data that is available through a fully searchable data dictionary and variable search tool.

Ethical approval for the study was obtained from the ALSPAC Ethics and Law Committee (ALEC; IRB00003312) and the Local Research Ethics Committees. Detailed information on the ways in which confidentiality of the cohort is maintained may be found on the study website.

Implied consent for the use of data collected via questionnaires was obtained from participants following the recommendations of the ALSPAC Ethics and Law Committee at the time ( Birmingham, 2018).

### The data

During pregnancy, as part of the questionnaire entitled “About Yourself” completed at about 12–14 weeks gestation ( Iles-Caven *et al.*, 2020) but occasionally postnatally as the questions were not time-sensitive, study women were asked to complete a diary of major events that occurred during their childhoods. The diary took the form of a table with 17 rows (one for each year of their life from <1 year old through to age 16 years) and three columns, in which they were instructed to record where they were living at that point (town and country), who was looking after them, and any major events that occurred during that year.

The diary was comprised entirely of free text and once the completed questionnaires were returned to ALSPAC, the responses were keyed into a delimited plain text file by a data entry clerk. The resulting file contained one row of data per respondent per year, each row containing the ID number of the respondent (which could not be linked back to their personal details), the age, and the three fields from the table: location, main carer(s), and major event.

In order to code the text data, the delimited file was imported into SPSS, where empty numeric variables were added to contain the codes that would be assigned. A total of 159,459 records were on file, from 11,754 respondents.

### Records

Most of the records on the file covered a single year for a single respondent, but there were some that spanned multiple years. Where this was the case, additional rows were created so that each one contained a single year of data, ensuring each row was uniquely identified by the combination of respondent ID and age. This increased the number of records from 159,459 to 174,056.

9752 of the 11,754 respondents (83.0%) completed all seventeen rows of the events diary, while the remaining 17.0% completed at least one row ( Table 1). Any rows that were entirely missing from the original delimited text file remained missing on the SPSS file. The most commonly completed row was Year 0 (equivalent to age <1 year), which was completed by 11,563 respondents (98.4% of the people who completed the events diary).

**Table 1.** Number of years for which childhood trauma data was provided by the women completing the “About Yourself” Questionnaire (N=12,448).

Number of diary rows completed	N (%)
0	694 (5.6)
1	366 (2.9)
2	375 (3.0)
3	327 (2.6)
4	234 (1.9)
5	193 (1.6)
6	99 (0.8)
7	206 (1.7)
8	49 (0.4)
9	41 (0.3)
10	24 (0.2)
11	13 (0.1)
12	13 (0.1)
13	10 (0.1)
14	7 (0.1)
15	10 (0.1)
16	35 (0.3)
17	9752 (78.3)
Completed diaries	11,754 (94.4)
Questionnaire completed	12,448

### Text coding

**Location**: The first piece of information requested was the town and country the respondents lived in during each year of their childhood. The majority of text responses (91.8%) were coded into the following categories: Avon, Rest of England, Wales, Scotland, and Northern Ireland. Other locations were coded, where possible, to the country where they lived using the International Standards Organisation numeric country codes (ISO 3166-1 numeric), with a further twenty codes added for responses that were not specific enough to fit into the ISO’s categories, or which covered multiple locations (N=6668).

A small number of respondents (3.7% of the responses given) appeared to misunderstand the request to supply the town and country where they lived, and instead wrote either “Town” or “Country” depending on whether they lived in an urban or rural area. We therefore do not assign a location and they were included in a category labelled “Non-geographic response”.

Where a location was given in one row, but not in the following ones, it was assumed there was no change in where the respondent was living, and the location code was copied down into the blank rows. This was because it was common for respondents to only note changes of location and to leave any rows blank when they did not move. The list of locations ( Table 2a) provides 127 valid codes.

**Table 2a.** First iteration of location coding, based on ISO 3166-1 numeric country codes with some additional codes.

Country/Territory	N	%
1 England: Avon area	101856	59.7
2 England: Not in Avon	47911	28.1
3 Wales	4035	2.4
4 Scotland	1950	1.1
5 Northern Ireland	696	0.4
12 Algeria	39	< 0.1
32 Argentina	<5	< 0.1
36 Australia	465	0.3
40 Austria	54	< 0.1
44 Bahamas	5	< 0.1
48 Bahrain	13	< 0.1
50 Bangladesh	73	< 0.1
52 Barbados	9	< 0.1
56 Belgium	34	< 0.1
60 Bermuda	<5	< 0.1
76 Brazil	20	< 0.1
96 Brunei	37	< 0.1
124 Canada	172	0.1
144 Sri Lanka	14	< 0.1
152 Chile	38	< 0.1
156 China	18	< 0.1
158 Taiwan	<5	< 0.1
170 Colombia	5	< 0.1
196 Cyprus	145	0.1
200 Czechoslovakia	17	< 0.1
208 Denmark	53	< 0.1
218 Ecuador	17	< 0.1
230 Ethiopia	<5	< 0.1
242 Fiji	<5	< 0.1
246 Finland	22	< 0.1
250 France	282	0.2
276 Germany/West Germany	785	0.5
278 East Germany	<5	< 0.1
288 Ghana	14	< 0.1
292 Gibraltar	53	< 0.1
300 Greece	54	< 0.1
328 Guyana	19	< 0.1
340 Honduras	<5	< 0.1
344 Hong Kong	272	0.2
356 India	191	0.1
360 Indonesia	22	< 0.1
364 Iran (Persia)	42	< 0.1
368 Iraq	20	< 0.1
372 Ireland	783	0.5
376 Israel	<5	< 0.1
380 Italy	64	< 0.1
388 Jamaica	208	0.1
392 Japan	22	< 0.1
400 Jordan	<5	< 0.1
404 Kenya	150	0.1
414 Kuwait	21	< 0.1
418 Laos	<5	< 0.1
422 Lebanon	<5	< 0.1
434 Libya	17	< 0.1
442 Luxembourg	14	< 0.1
450 Madagascar	10	< 0.1
458 Malaya/Malaysia	143	0.1
470 Malta	103	0.1
480 Mauritius	54	< 0.1
484 Mexico	21	< 0.1
504 Morocco	38	< 0.1
512 Oman	<5	< 0.1
524 Nepal	5	< 0.1
528 Netherlands	185	0.1
554 New Zealand	210	0.1
566 Nigeria	88	0.1
578 Norway	48	< 0.1
586 Pakistan	97	0.1
598 Papua New Guinea	9	< 0.1
608 Philippines	136	0.1
616 Poland	34	< 0.1
620 Portugal	29	< 0.1
634 Qatar	14	< 0.1
642 Romania	9	< 0.1
654 St Helena	<5	< 0.1
659 St Kitts & Nevis	11	< 0.1
682 Saudi Arabia	22	< 0.1
686 Senegal	7	< 0.1
690 Seychelles	6	< 0.1
702 Singapore	190	0.1
704 Vietnam	33	< 0.1
706 Somalia	<5	< 0.1
710 South Africa	216	0.1
716 Zimbabwe (Rhodesia)	126	0.1
720 Yemen (Aden)	31	< 0.1
724 Spain	108	0.1
729 Sudan	8	< 0.1
748 Eswatini (Swaziland)	<5	< 0.1
752 Sweden	31	< 0.1
756 Switzerland	26	< 0.1
760 Syria	17	< 0.1
764 Thailand	48	< 0.1
780 Trinidad & Tobago	39	< 0.1
784 United Arab Emirates (Trucial States)	<5	< 0.1
792 Turkey	<5	< 0.1
800 Uganda	65	< 0.1
810 Soviet Union	<5	< 0.1
818 Egypt	18	< 0.1
826 United Kingdom (constituent country not known)	97	0.1
831 Guernsey	71	< 0.1
832 Jersey	26	< 0.1
833 Isle of Man	46	< 0.1
834 Tanzania	58	< 0.1
840 USA	395	0.2
862 Venezuela	9	< 0.1
890 Yugoslavia	36	< 0.1
894 Zambia	88	0.1
900 Moved around	71	< 0.1
901 Various military bases	40	< 0.1
950 East Africa	16	< 0.1
951 Africa	12	< 0.1
952 West Africa	15	< 0.1
953 Caribbean	11	< 0.1
954 Arabia	10	< 0.1
955 Overseas	5	< 0.1
990 Non-geographical response	118	0.1
995 Response illegible	66	< 0.1
997 Country (area not known)	2457	1.4
998 Town (area not known)	3817	2.2
1276 Avon and Germany	6	< 0.1
2276 England and Germany	<5	< 0.1
2833 England and Isle of Man	6	< 0.1
3422 Wales and Lebanon	<5	< 0.1
36598 Australia & Papua New Guinea	<5	< 0.1
192208 Cuba and Denmark	<5	< 0.1
826404 UK and Kenya	<5	< 0.1
826458 UK and Malaya/Malaysia	5	< 0.1
**Total**	**170,509**	**100.0**

In order to minimise identification, a new variable was derived which categorised the list of countries based on global region. The original locations of Avon, rest of England, Wales, Scotland, and Northern Ireland were retained, while the others were grouped together as shown in Table 2b. Respondents who stated they lived in the UK but were not more specific were categorised with respondents from the Isle of Man and the Channel Islands (collectively known as the Crown Dependencies). The rest of Europe was divided into two groups, Western Europe and Eastern Europe, the latter of which contained the former Eastern Bloc nations with the remainder of the continent in the former category.

**Table 2b.** Coding of respondent’s location and number of diary rows per location.

Location code	Country/region code(s) included	N (valid %)
1: Avon	Avon	101856 (62.1)
2: Rest of England	Rest of England	47911 (29.2)
3: Wales	Wales	4035 (2.5)
4: Scotland	Scotland	1950 (1.2)
5: Northern Ireland	Northern Ireland	696 (0.4)
6: UK & Crown Dependencies	United Kingdom (constituent country not stated); Guernsey; Jersey; Isle of Man; England and Isle of Man	246 (0.1)
7: Western Europe	Austria; Belgium; Cyprus; Denmark; Finland; France; Germany/West Germany; Gibraltar; Greece; Ireland; Italy; Luxembourg; Malta; Netherlands; Norway; Portugal; Spain; Sweden; Switzerland	2873 (1.8)
8: Eastern Europe	Czechoslovakia; East Germany; Poland; Romania; Soviet Union; Yugoslavia	101 (0.1)
9: West Africa	Ghana; Nigeria; Senegal; West Africa	124 (0.1)
10: North Africa	Algeria; Libya; Morocco; Egypt	112 (0.1)
11: East Africa	Ethiopia; Kenya; Madagascar; Mauritius; Seychelles; Somalia; Sudan; Tanzania; Uganda; East Africa	375 (0.2)
12: Southern Africa	South Africa; Zimbabwe (Rhodesia); Kingdom of Eswatini (Swaziland); Zambia	433 (0.3)
13: West Asia/Middle East	Bahrain; Iran (Persia); Iraq; Israel; Jordan; Kuwait; Lebanon; Oman; Qatar; Saudi Arabia; Yemen (Aden); Syria; United Arab Emirates (Trucial States); Turkey; Arabia	208 (0.1)
14: South Asia	Bangladesh; Sri Lanka; India; Nepal; Pakistan	380 (0.2)
15: South-East Asia	Brunei; Indonesia; Laos; Malaya/Malaysia; Philippines; Singapore; Vietnam; Thailand	611 (0.4)
16: East Asia	China; Taiwan; Hong Kong; Japan	313 (0.2)
17: Oceania	Australia; Fiji; New Zealand; Papua New Guinea; Australia and Papua New Guinea	688 (0.4)
18: North America	Canada; Mexico; USA	588 (0.4)
19: South/Central America	Argentina; Brazil; Chile; Colombia; Ecuador; Guyana; Honduras; Venezuela	113 (0.1)
20: Caribbean/ Atlantic Islands	Bahamas; Barbados; Bermuda; Jamaica; St Helena; St Kitts & Nevis; Trinidad & Tobago; Caribbean	289 (0.2)
21: Moved around/multiple regions	Moved around; Various military bases; Africa; Avon & Germany; England & Germany; Wales & Lebanon; Cuba & Denmark; UK & Kenya; UK & Malaysia	144 (0.1)
*Set to missing*	*Overseas; Non-geographic response; Response illegible; Country; Town*	*6463*
**Valid total**		**164,046**

Africa was divided into four areas: West, North, East, and Southern, while Asian countries were categorised as one of West Asia/Middle East, South Asia, South-East Asia, or East Asia. Australia, New Zealand and the island nations of the Pacific were grouped together as Oceania, and the Americas were categorised into North, South/Central, and Caribbean/Atlantic islands.

A further category was added for people who stated that they had moved around or lived in multiple locations in a single year. Where no useful geographic location was given, these were set to missing.

After the completion of the coding process, the data file was reshaped so that there was a single row of data per respondent. This decreased the number of rows from 174,056 to 11,754 and increased the number of variables from six to 86.

Table 2c shows the frequencies of the location categories, and the names of the variables, following the reshaping of the data file.

**Table 2c.** Frequencies of locations of respondents during each year of their childhood.

Mother’s age (years)	<1	1	2	3	4	5	6	7	8	9	10	11	12	13	14	15	16
Variable name	d900	d905	d910	d915	d920	d925	d930	d935	d940	d945	d950	d955	d960	d965	d970	d975	d980
Avon	6118	5389	5497	5597	5720	5813	5850	5929	5955	6000	6100	6197	6244	6294	6310	6365	6478
Rest of England	3651	3070	3083	2973	2917	2893	2836	2797	2768	2724	2699	2677	2611	2592	2561	2529	2530
Wales	307	263	242	246	235	230	232	230	224	231	237	232	227	229	229	222	219
Scotland	157	133	134	130	129	123	122	123	118	112	103	96	96	94	95	94	91
N. Ireland	52	44	47	42	39	43	43	40	41	38	40	41	41	38	35	37	35
UK (NOS)/Crown dependencies	9	10	10	10	13	16	14	13	14	15	14	13	16	17	19	22	21
Western Europe	228	195	194	207	207	202	186	169	158	165	159	154	147	137	132	122	111
Eastern Europe	8	6	5	5	5	5	7	7	7	7	6	6	5	5	5	6	6
West Africa	14	15	15	11	8	8	6	6	6	6	7	<5	<5	<5	<5	<5	<5
North Africa	12	9	8	10	7	6	6	6	6	5	6	6	5	5	5	5	5
East Africa	45	34	32	33	28	25	18	19	18	21	22	21	19	14	9	9	8
Southern Africa	35	32	37	37	31	27	26	25	27	26	23	23	20	15	16	15	18
West Asia	20	20	18	18	13	11	10	13	10	13	9	11	8	10	7	8	9
South Asia	33	30	32	29	26	27	26	22	21	17	17	17	18	16	17	16	16
South-East Asia	55	48	51	52	45	42	39	32	34	34	30	32	29	23	23	21	21
East Asia	25	23	20	19	20	20	22	22	20	20	17	15	15	13	14	16	12
Oceania	38	35	39	42	45	48	47	57	52	46	48	44	34	28	27	28	30
North America	47	40	43	43	39	36	38	39	35	37	30	27	31	31	25	25	22
S./C. America	11	10	8	7	8	8	8	7	6	6	7	6	5	<5	<5	<5	<5
Caribbean/Atlantic	27	24	27	26	26	26	20	20	16	15	12	9	8	9	8	8	8
Multiple regions	12	11	12	12	12	10	7	8	8	8	6	<5	7	7	6	6	8
**Total**	**10904**	**9441**	**9554**	**9549**	**9573**	**9619**	**9563**	**9584**	**9544**	**9546**	**9592**	**9634**	**9590**	**9585**	**9551**	**9562**	**9655**

**Carer**: As with location, a long list of possible values was gleaned from the text data and codes were assigned according to which of these categories the carer (or carers) fitted. This first iteration of codes, the full list of which can be seen in Table 3a, resulted in 68 possible values. These 68 categories were consolidated into 11 groups described in Table 3b along with the total number of diary rows per carer type. The frequencies and variable names for carers during each year of childhood are shown in Table 3c.

**Table 3a.** Initial coding of respondents’ carers during childhood.

Main carer(s)	N	%
Mother & father	119995	70.1
Mother	35798	20.9
Father	1526	0.9
Grandmother & grandfather	399	0.2
Grandmother	797	0.5
Grandfather	20	< 0.1
Mother & stepfather	3529	2.1
Father & stepmother	652	0.4
Sister	127	0.1
Brother	49	< 0.1
Mother, father & other family	1963	1.1
Aunt & uncle	62	< 0.1
Aunt	145	0.1
Uncle	17	< 0.1
Other family member	<5	< 0.1
Mother & other family	1776	1.0
Father & other family	335	0.2
Family (other combination or not specified)	165	0.1
Adoptive parents	795	0.5
Adoptive mother	70	< 0.1
Adoptive father	<5	< 0.1
Foster parents	245	0.1
Foster mother	40	< 0.1
Children's home/orphanage/convent	289	0.2
In care	133	0.1
Guardian	25	< 0.1
Adoptive father & stepmother	15	< 0.1
Nanny/maid/staff	89	0.1
Parents & nanny/maid/staff	232	0.1
Mother & nanny/maid/staff	304	0.2
Father & nanny/maid/staff	21	< 0.1
Boarding school	315	0.2
Parents & school	234	0.1
Hospital/health authority	9	< 0.1
Nursing home/hospital & mother	<5	< 0.1
Mother & school/nursery	123	0.1
Parents & hospital	8	< 0.1
Friend's parent(s)	25	< 0.1
Family friend(s)	40	< 0.1
Godparents	<5	< 0.1
Mother & friend(s)	60	< 0.1
Stepmother	130	0.1
Mother, stepfather & other family	39	< 0.1
Father & friend(s)	12	< 0.1
Stepfather	44	< 0.1
Mother, stepfather & friend(s)	<5	< 0.1
Parents & friend(s)	38	< 0.1
No one	416	0.2
Various people	12	< 0.1
In and out of care	<5	< 0.1
Sister and self	<5	< 0.1
Foster parent	8	< 0.1
Stepparent	<5	< 0.1
Mother, nanny & other family	<5	< 0.1
Father, stepmother & other family	10	< 0.1
Aunt, uncle & neighbours	<5	< 0.1
Mother figure & father figure	17	< 0.1
Parents, other family & nanny/maid/staff	21	< 0.1
Mother & godmother	<5	< 0.1
Parents & someone else	25	< 0.1
Sister & boarding school	<5	< 0.1
Nursery school	<5	< 0.1
Father, other family & nanny/maid/staff	<5	< 0.1
Stepmother & school	<5	< 0.1
School & nanny/maid/staff	<5	< 0.1
Father & school	<5	< 0.1
Other	36	< 0.1
Response illegible	17	< 0.1
**Total**	**171,299**	**100.0**

**Table 3b.** Coding of respondent’s carers during childhood and number of diary rows per carer type.

Carer	Carer(s) named by respondent	N (valid %)
Mother & father	Mother & father; Mother, father & other family; Parents & nanny/maid/staff; Parents & school; Parents & hospital; Parents & friend(s); Parents, other family & nanny/maid/staff; Parents & someone else	122516 (71.5)
Mother	Mother; Mother & other family; Mother & nanny/maid/staff; Nursing home/hospital & mother; Mother & school/nursery; Mother & friend(s); Mother, nanny & other family; Mother & godmother	38068 (22.2)
Father	Father; Father & other family; Father & nanny/maid/staff; Father & friends; Father, other family & nanny/maid/staff; Father & school	1900 (1.1)
Mother & stepfather	Mother & stepfather; Mother, stepfather & other family; Stepfather; Mother, stepfather & friend(s)	3615 (2.1)
Father & stepmother	Father & stepmother; Stepmother; Father, stepmother & other family; Stepmother & school	793 (0.5)
Other family member(s)	Grandmother & grandfather; Grandmother; Grandfather; Sister; Brother; Aunt & uncle; Aunt; Uncle; Other family member; Family; Sister & self; Aunt, uncle & neighbours; Sister & boarding school	1792 (1.0)
Adoptive/foster parent(s)	Adoptive parents; Adoptive mother; Adoptive father; Foster parents; Foster mother; Guardian; Adoptive father & stepmother; Foster parent; Stepparent; Mother figure & father figure	1221 (0.7)
In care	Children’s home/orphanage/convent; In care; In and out of care	426 (0.2)
Paid childcare	Nanny/maid/staff; Boarding school; Hospital/health authority; Nursery school; School & nanny/maid/staff	418 (0.2)
Other people	Friend’s parent(s); Family friend(s); Godparents; Various people; Other	117 (0.1)
Nobody	No one	416 (0.2)
*Set to missing*	*Illegible*	*17*
**Valid total**		**171,282**

**Table 3c.** Frequencies of main carers of respondents during each year of their childhoods.

Mother’s age (year)	<1	1	2	3	4	5	6	7	8	9	10	11	12	13	14	15	16
**Variable name**	**d901**	**d906**	**d911**	**d916**	**d921**	**d926**	**d931**	**d936**	**d941**	**d946**	**d951**	**d956**	**d961**	**d966**	**d971**	**d976**	**d981**
Mother & Father	8666	7475	7568	7514	7524	7465	7349	7310	7188	7126	7090	6993	6846	6745	6632	6537	6488
Mother	2546	2173	2157	2160	2124	2161	2133	2153	2175	2170	2209	2258	2287	2297	2333	2358	2374
Father	20	22	33	42	55	69	80	82	100	115	126	154	168	194	208	218	214
Mother & stepfather	9	17	31	53	76	118	159	195	225	259	295	329	360	368	371	379	371
Father & stepmother	<5	<5	<5	5	15	20	29	38	45	59	65	69	82	89	93	94	86
Other family	89	98	110	106	110	112	116	110	112	105	93	96	97	99	105	116	118
Adoptive/foster parents	39	53	59	64	66	69	67	72	74	77	77	80	78	87	84	88	87
In care	12	12	15	16	17	15	19	23	27	25	27	25	27	33	43	49	41
Paid childcare	12	13	13	14	10	7	14	9	11	16	16	35	49	54	51	50	44
Other	5	6	5	<5	<5	<5	5	6	6	<5	5	5	<5	8	13	15	21
Nobody	<5	-	-	-	-	-	-	-	-	<5	<5	9	17	26	42	77	238
**Total**	**11400**	**9870**	**9993**	**9976**	**10000**	**10040**	**9971**	**9998**	**9963**	**9958**	**10007**	**10053**	**10015**	**10000**	**9975**	**9981**	**10082**

**Event**: The final piece of information requested in the diary was any major event(s), that happened to the respondent in each year of her childhood. The responses to this were wide-ranging and coding again took place in two steps.

Events that were recorded by the mother were first categorised into one of 225 categories, with the exception of events that occurred to all (or virtually all) of the respondents such as starting school, or events considered to be non-traumatic that were noted, such as going on holiday or riding a horse. These events were not assigned codes for the purposes of this work. The initial categorisation was undertaken by SG.

Because some respondents noted multiple events (maximum recorded: 3) in a single year, up to three event variables were added to the data file to record these. All these variables used the same coding system.

Once the initial coding of these events was finalised, they were re-assigned to broad categories to minimise disclosure risk. These categories were defined according to the amount of trauma that was likely to have been caused to the respondent:

1. Bereavement in immediate family (parent/sibling)
2. Other bereavement
3. Severe trauma or in traumatic situation
4. Moderate trauma
5. Mild trauma
6. Probably not traumatic to study participant

This categorisation was performed by a panel of five coders (SG, JG, YIC and two others – see Acknowledgements) who were blind to each other’s responses. Each of the coders went through all 225 of the event codes and assigned them a value based on the amount of trauma they felt that event would cause. These five sets of codes were then compared, and where there was a majority agreement (three or more coders concurring) on the trauma level caused, this was the value assigned. Where there was no majority agreement the median of the trauma levels assigned by the coders was used. A combined frequency table for all three of these variables, along with the level of trauma category assigned by each coder, and the final category assigned, is shown in Table 4. Based on the consensus between coders, the severity of trauma frequencies is shown in Table 5a, along with the order in which the event was recorded in the diary. The frequencies after reshaping the data into the final data file are shown in Table 5b.

**Table 4.** Events that occurred during the respondents’ childhood. The list of potentially traumatic events given by respondents in the childhood events diary is shown here along with the category assigned to each one by each of the five coders. Where there was majority agreement on a category between the coders this was used, otherwise the median of the coders’ categories was used. The categories are 1=Bereavement in immediate family, 2=Other bereavement, 3=Severe trauma, 4=Moderate trauma, 5=Mild trauma, 6=Probably not traumatic.

Event	N	Coder 1	Coder 2	Coder 3	Coder 4	Coder 5	Category assigned
Parent died	599	1	1	1	1	1	**1**
Sibling died	160	1	1	1	1	1	**1**
Parent committed suicide	8	1	1	1	1	1	**1**
Sibling committed suicide	<5	1	1	1	1	1	**1**
Grandparent died	2783	2	1	1	2	2	**2**
Another family member or friend died	429	2	1	1	2	2	**2**
Pet died	45	2	2	2	2	5	**2**
Death, person not specified	142	2	2	2	2	2	**2**
Suffered serious injury	369	4	3	3	3	3	**3**
Parent injured in terrorist attack	<5	3	3	3	3	3	**3**
Other relation injured in terrorist attack	<5	4	3	3	3	3	**3**
Suffered serious illness	278	3	3	3	4	3	**3**
Parent had serious illness	411	4	4	3	3	3	**3**
Had a serious accident	305	4	3	3	4	3	**3**
Parent had a serious accident	88	4	3	3	3	3	**3**
Sibling had a serious accident	71	4	3	3	4	3	**3**
Taken into care	67	3	3	3	3	3	**3**
Thrown out of family home	8	3	3	3	3	3	**3**
Own child given up for adoption	<5	4	3	3	4	3	**3**
Made to have termination of pregnancy by parents	<5	3	3	3	4	3	**3**
Sexually abused	142	3	3	3	3	3	**3**
Physically abused/beaten	53	3	3	3	3	3	**3**
Abused (nature of abuse not specified)	49	3	3	3	3	3	**3**
Raped	17	3	3	3	3	3	**3**
Sexually assaulted	26	3	3	3	3	3	**3**
Assaulted	6	4	3	3	3	3	**3**
Threatened with a weapon	<5	3	3	3	3	4	**3**
War/revolution/coup	25	3	3	3	4	3	**3**
Long-term condition diagnosed	60	4	3	3	4	3	**3**
Attempted suicide	5	3	3	3	3	3	**3**
Abandoned/left by parent	21	3	3	3	3	3	**3**
Birth trauma	<5	6	3	3	6	3	**3**
Sent to prison	<5	3	4	3	4	3	**3**
Overdosed	24	3	3	3	3	3	**3**
Parent attempted suicide	10	3	3	3	3	3	**3**
Alcoholism/alcohol abuse	<5	4	3	3	3	4	**3**
Other family member/friend committed suicide	9	2	4	3	4	3	**3**
Parent was alcoholic/drank heavily	33	4	4	3	3	3	**3**
Grandparent committed suicide	<5	2	3	3	4	3	**3**
Parent took overdose	6	3	3	3	4	3	**3**
Partially blinded	<5	4	3	3	4	3	**3**
Parent shot	<5	3	3	3	3	3	**3**
Sibling attempted suicide	<5	4	3	3	4	3	**3**
Shipwrecked	<5	4	3	3	4	3	**3**
Had nervous breakdown	<5	3	3	3	3	3	**3**
Parent raped	<5	3	3	3	4	3	**3**
Ingested poisonous substance	<5	5	3	3	3	3	**3**
Sibling raped	<5	3	4	3	4	3	**3**
Best/close friend died	<5	2	3	3	2	3	**3**
Became homeless	8	3	3	3	4	3	**3**
Mother beaten by father	14	3	3	3	3	3	**3**
Grandparents tried taking respondent from parents	<5	4	3	3	4	3	**3**
Father tried to kill mother	<5	3	3	3	3	3	**3**
Saw parents fighting with a weapon	<5	4	3	3	3	3	**3**
Caught in a building fire	<5	3	3	3	3	3	**3**
Attempted sexual assault/abuse	5	3	4	3	3	3	**3**
Parent attempted to kill respondent	<5	3	3	3	3	3	**3**
Witnessed a murder	<5	3	3	3	3	3	**3**
Parent accused of abuse by another child	<5	4	3	3	4	3	**3**
Born with a cleft palate	<5	4	3	3	6	3	**3**
Caught having sex with brother	<5	3	3	3	4	3	**3**
Found body of deceased grandparent	<5	4	3	3	3	3	**3**
Sibling had serious illness	52	4	4	3	4	3	**4**
Grandparent had serious illness	24	4	4	3	4	4	**4**
Other family member had serious illness	<5	4	4	3	4	4	**4**
Parent suffered serious injury	18	5	4	4	3	3	**4**
Sibling suffered serious injury	11	5	4	4	4	3	**4**
Hospitalised	720	4	3	4	5	4	**4**
Parent hospitalised	266	5	3	4	5	4	**4**
Sibling hospitalised	27	5	3	4	5	4	**4**
Grandparent had a serious accident	7	5	3	3	4	4	**4**
Someone else had a serious accident	<5	5	4	5	4	4	**4**
Parent lost job	43	4	4	4	5	4	**4**
Parents divorced/separated	1589	3	4	4	4	3	**4**
Parents had marital problems	146	4	5	4	4	5	**4**
Mother had a miscarriage/stillbirth	33	4	4	4	3	5	**4**
Ran away from home	24	4	3	3	4	4	**4**
Placed with foster parents	22	5	4	4	4	4	**4**
Bullied at school	30	4	3	3	4	4	**4**
Unhappy at school and/or home	112	5	4	4	4	5	**4**
Sibling ran away from home	5	5	3	3	4	5	**4**
Fire or flood in home	22	4	3	3	4	4	**4**
Main carer for ill parent	<5	4	4	4	3	5	**4**
Main carer for sibling(s)	8	4	5	4	3	5	**4**
Sibling born with disability	15	5	4	4	5	4	**4**
Fighting/arguing with parent(s)	25	5	4	4	4	5	**4**
Conflict/arguments at home	18	5	4	4	4	5	**4**
Family business failed	<5	5	4	4	4	4	**4**
Became pregnant	85	4	4	4	4	6	**4**
Had a termination of pregnancy	30	4	4	4	4	4	**4**
Had a miscarriage	5	4	3	3	4	4	**4**
Own child born	26	5	4	4	3	6	**4**
Ectopic pregnancy	<5	5	4	4	4	4	**4**
Physically interfered with	<5	4	3	3	4	4	**4**
Eating disorder	17	4	3	3	4	4	**4**
Depression	6	4	4	4	4	4	**4**
Parent had depression	29	5	4	4	4	4	**4**
In trouble with police	<5	4	4	4	3	4	**4**
Parent sent to prison	27	3	4	4	4	3	**4**
Sibling sent to prison	<5	3	4	4	5	3	**4**
Drug use	<5	4	4	4	6	5	**4**
Parent became disabled	<5	4	3	4	5	3	**4**
Parent had nervous breakdown	65	4	3	4	4	3	**4**
Involved in court case	7	4	5	4	5	4	**4**
Involved in custody hearing	9	4	5	4	4	4	**4**
Parent diagnosed with long-term illness	34	5	3	4	4	3	**4**
Parent in psychiatric hospital	22	4	3	4	4	3	**4**
Sibling diagnosed with long-term illness	9	5	4	4	5	3	**4**
Sibling self-harmed	<5	5	4	3	4	4	**4**
Locked up by parent	<5	4	4	4	3	3	**4**
Sibling became disabled	<5	4	4	4	4	3	**4**
Sibling had eating disorder	<5	5	4	4	3	4	**4**
Sibling fighting in war	<5	5	4	4	4	3	**4**
Required psychiatric treatment	<5	4	3	4	3	4	**4**
Bed wetting	6	5	4	4	5	4	**4**
Sibling in trouble with police	<5	5	4	4	4	5	**4**
Parent had mental illness	5	5	4	4	4	5	**4**
Sibling had nervous breakdown	7	5	4	4	4	5	**4**
Parent was agoraphobic	<5	5	5	4	4	4	**4**
Parent fighting in war	<5	4	4	4	3	3	**4**
Parent attacked in street	<5	4	4	4	4	3	**4**
Grandparent had nervous breakdown	10	5	4	4	4	4	**4**
Unwanted sexual attention	<5	4	5	5	4	4	**4**
Self-harm	<5	4	3	4	4	4	**4**
Drug use by sibling	<5	5	4	4	3	5	**4**
Family evicted from home	7	4	4	4	4	3	**4**
Expelled from school	12	4	6	4	5	4	**4**
Sibling had a stillbirth	<5	4	3	4	5	6	**4**
Found out she was adopted	<5	5	4	4	5	4	**4**
Parent very unhappy	<5	5	4	4	5	4	**4**
Got divorced	<5	4	4	4	4	4	**4**
House burgled	<5	4	3	3	5	4	**4**
Not seen parent after this age	<5	4	4	4	3	4	**4**
Prevented from seeing parent	5	4	4	4	3	4	**4**
Deaf/hearing impairment	7	5	4	3	4	4	**4**
Reduced mobility	5	5	4	4	5	4	**4**
Sent to live away from home	<5	5	4	4	4	4	**4**
Scared of father	9	5	4	4	4	4	**4**
Forced to eat meat	<5	4	4	4	4	4	**4**
Respondent thought she was pregnant	<5	5	4	4	5	4	**4**
Forced by parents to leave school	<5	5	4	5	4	4	**4**
Sister's baby taken away by its father	<5	5	3	5	4	4	**4**
Another person suffered serious injury	<5	5	5	4	5	4	**5**
Serious illness, person not specified	17	4	5	5	4	5	**5**
Grandparent hospitalised	<5	5	3	4	5	5	**5**
Unspecified person hospitalised	15	5	4	5	6	5	**5**
Serious accident (victim(s) not specified)	49	5	4	5	4	5	**5**
Parent re-married or moved in with new partner	519	4	6	5	4	5	**5**
New sibling born (incl. half-siblings)	2103	6	5	5	3	6	**5**
Another child moved into family home	72	5	5	5	5	5	**5**
Another adult moved into family home	69	5	5	5	5	6	**5**
Adopted	44	5	5	5	3	3	**5**
Moved home	2034	6	5	6	5	5	**5**
Changed schools (excl. primary to secondary)	474	6	5	6	5	5	**5**
Sent to boarding school	223	5	4	5	4	6	**5**
Parent(s) absent from home	177	6	5	5	5	6	**5**
Left home	212	6	5	6	4	5	**5**
Parents divorced (already separated)	113	5	6	5	5	4	**5**
Failed exam(s)	14	5	4	4	5	5	**5**
Family moved into someone else's home	39	5	5	5	5	4	**5**
Mother had a termination of pregnancy	<5	5	4	4	5	5	**5**
Other family member/friend sent to prison	<5	5	5	5	5	4	**5**
Parent on medication	<5	6	4	5	5	6	**5**
Diagnosed with dyslexia	<5	6	5	5	6	5	**5**
In trouble at home/school	11	5	5	4	5	5	**5**
Truancy	<5	6	6	5	5	5	**5**
Family had financial difficulties	12	6	5	4	5	5	**5**
Divorce, people not specified	36	5	4	5	4	6	**5**
Became responsible for looking after home	<5	4	5	5	4	5	**5**
Sister had a termination of pregnancy	<5	5	4	4	5	5	**5**
Helped care for infirm relative	<5	5	5	5	3	6	**5**
Sibling divorced	11	5	5	5	5	5	**5**
Grandparents divorced	<5	5	5	4	5	5	**5**
Reunited with estranged parent	28	6	5	5	4	5	**5**
Prevented from seeing other family member	<5	5	5	5	4	5	**5**
Flashed at	<5	5	5	5	5	5	**5**
Received counselling	<5	6	5	5	3	6	**5**
Sibling had child with disability	<5	5	5	5	5	4	**5**
Separated from sibling	<5	5	4	5	5	6	**5**
Discovered existence of half sibling	<5	5	5	5	5	4	**5**
Other family member had mental health problem	<5	5	5	5	5	5	**5**
Parent met new partner	10	5	6	5	5	6	**5**
Locked self in small room	<5	5	4	5	5	5	**5**
Lived in high crime area	<5	4	4	5	5	6	**5**
Friend moved away	<5	5	5	5	4	6	**5**
Moved up a year at school	<5	5	6	6	5	5	**5**
Started wearing glasses	<5	5	4	5	3	6	**5**
Discovered parents weren't married	<5	5	5	6	5	5	**5**
Father in armed forces	6	5	4	5	6	6	**5**
Sent to different school from friends	<5	5	4	5	5	5	**5**
Change of house parents at children's home	<5	5	4	5	5	6	**5**
Left alone when ill by au pair	<5	5	4	4	5	5	**5**
Discovered grandparent who was thought dead was still alive	<5	6	5	5	5	5	**5**
Discovered sibling was adopted	<5	5	5	5	4	6	**5**
Parents re-united after splitting up	92	5	6	6	6	6	**6**
Parent started/returned to work	48	6	6	6	6	6	**6**
Parent left work by choice	24	6	6	6	6	6	**6**
Started work	138	5	6	6	5	6	**6**
Changed jobs	<5	6	6	6	5	6	**6**
Sibling married/left home	285	6	6	6	5	6	**6**
Parent started own business	11	6	6	5	6	6	**6**
Sibling had a baby	49	6	6	6	6	6	**6**
Married	23	6	6	6	6	6	**6**
Became engaged	8	6	6	6	6	6	**6**
Boyfriend moved into family home	<5	6	6	5	6	5	**6**
Saw child psychologist	<5	6	5	5	6	6	**6**
Sibling joined military	<5	5	6	5	6	6	**6**
Birth (person not specified)	<5	6	6	6	?	6	**6**
Pregnancy: person not stated	<5	6	6	6	6	6	**6**
Sibling became pregnant	<5	6	6	6	6	6	**6**
Grandparent re-married	7	6	6	6	6	6	**6**
Parent married partner (already co-cohabiting)	<5	6	6	6	5	6	**6**
Sibling went to boarding school	5	6	6	5	5	6	**6**
Other child moved out of home	<5	6	6	6	6	6	**6**
Other adult moved out of home	<5	6	6	6	6	6	**6**
Sibling had problems at home/school	8	6	5	6	5	6	**6**
Baptised/changed religion	6	6	6	6	6	6	**6**
Legally adopted by father-figure	6	6	6	5	6	6	**6**
Lived in children's home where parents worked	<5	5	4	6	6	6	**6**
Given alcohol by parents	<5	5	5	6	6	6	**6**
Other family member divorced	8	6	5	6	5	6	**6**
Parent stopped drinking alcohol	<5	6	6	6	6	6	**6**
Parents got married	<5	6	6	6	6	6	**6**
Allergy discovered	<5	6	5	5	6	6	**6**
Home move implied by other answers	7090	6	5	5	6	6	**6**

**Table 5a.** Frequencies of traumatic events categorised according to severity.

	First event	Second event	Third event	Total (%)
1: Bereavement in immediate family	736	30	2	768 (3.2)
2: Other bereavement	3145	230	23	3398 (14.0)
3: Severe trauma	1997	139	13	2149 (8.9)
4: Moderate trauma	3516	175	29	3720 (15.4)
5: Mild trauma	5774	521	38	6333 (26.2)
6: Probably not traumatic	6805	972	54	7831 (32.4)

**Table 5b.** Frequencies of the category of trauma for 1-3 events that happened to respondents in each year of their childhood.

Woman’s age	<1	1	2	3	4	5	6	7	8	9	10	11	12	13	14	15	16
FIRST EVENT																	
**Variable name**	**d902**	**d907**	**d912**	**d917**	**d922**	**d927**	**d932**	**d937**	**d942**	**d947**	**d952**	**d957**	**d962**	**d967**	**d972**	**d977**	**d982**
None	2357	1956	1870	1852	1859	1793	1855	1864	1863	1866	1825	1757	1818	1834	1826	1814	1704
1: Bereavement in immediate family	20	14	31	35	27	35	32	36	48	34	43	58	44	66	73	67	74
2: Other bereavement	39	31	68	71	102	133	153	187	196	179	262	301	282	292	281	269	300
3: Severe trauma	104	61	106	99	96	109	102	139	114	120	150	161	133	141	130	111	121
4: Moderate trauma	123	75	150	178	195	220	215	241	195	183	217	270	229	238	260	264	270
5: Mild trauma	145	217	559	473	415	433	363	359	351	280	358	382	327	294	235	233	360
6: Probably not traumatic	23	519	622	660	603	574	463	421	402	358	371	371	359	265	244	220	341
**Total**	**2811**	**2873**	**3406**	**3368**	**3297**	**3297**	**3183**	**3247**	**3169**	**3020**	**3226**	**3300**	**3192**	**3130**	**3049**	**2978**	**3170**
SECOND EVENT																	
**Variable name**	**d903**	**d908**	**d913**	**d918**	**d923**	**d928**	**d933**	**d938**	**d943**	**d948**	**d953**	**d958**	**d963**	**d968**	**d973**	**d978**	**d983**
None	-	<5	<5	-	<5	-	-	<5	-	-	-	<5	<5	<5	<5	<5	<5
1: Bereavement in immediate family	<5	-	<5	<5	<5	<5	<5	<5	<5	<5	-	<5	<5	<5	<5	-	<5
2: Other bereavement	7	<5	8	7	9	15	10	15	15	11	11	12	19	14	20	24	32
3: Severe trauma	<5	<5	5	8	10	<5	5	7	10	9	10	<5	9	23	12	12	8
4: Moderate trauma	5	<5	<5	10	7	13	9	10	7	9	9	18	12	10	11	15	25
5: Mild trauma	<5	7	30	30	28	34	39	54	41	28	45	40	37	27	29	28	24
6: Probably not traumatic	<5	27	67	73	71	79	76	68	72	59	56	61	55	60	56	41	50
**Total**	**22**	**38**	**117**	**131**	**130**	**145**	**141**	**159**	**148**	**119**	**131**	**138**	**134**	**139**	**130**	**121**	**143**
THIRD EVENT																	
**Variable name**	**d904**	**d904**	**d914**	**d919**	**d924**	**d929**	**d934**	**d939**	**d944**	**d949**	**d954**	**d959**	**d964**	**d969**	**d974**	**d979**	**d984**
None	-	-	<5	-	<5	-	-	<5	-	-	-	<5	<5	<5	<5	<5	<5
1: Bereavement in immediate family	<5	-	-	-	-	<5	-	-	-	-	-	-	-	-	-	-	-
2: Other bereavement	-	-	<5	-	<5	<5	<5	<5	-	<5	<5	<5	<5	-	<5	<5	7
3: Severe trauma	-	-	-	-	<5	-	<5	-	<5	<5	-	-	-	<5	<5	-	<5
4: Moderate trauma	<5	-	<5	<5	<5	<5	<5	-	<5	-	6	<5	<5	<5	<5	<5	5
5: Mild trauma	-	-	-	<5	<5	<5	<5	<5	5	<5	7	<5	5	-	<5	<5	-
6: Probably not traumatic	-	<5	<5	5	<5	5	<5	<5	<5	5	<5	<5	<5	5	<5	<5	<5
**Total**	**<5**	**<5**	**6**	**9**	**11**	**11**	**9**	**9**	**11**	**8**	**17**	**12**	**13**	**10**	**12**	**8**	**21**

## Strengths and limitations

A key strength of the data is its size, with 11,754 respondents reporting 24,199 different events, 16,368 of which were considered to have caused some level of trauma. As part of a major longitudinal study which has been running for over thirty years there has been a great deal of follow-up data collected on the original respondents, as well as on their parents and grandparents, their partners, their offspring, and now their grandchildren, providing huge potential for analyses on the antecedents and outcomes of childhood trauma across multiple generations.

A limitation of the data is that the level of trauma caused by any event is very subjective, so the same event may cause a great deal of distress to one individual but very little to another. Only the nature of each event was recorded; the respondents were not asked how much effect each event had on them. In addition, the data were collected retrospectively; for the older mothers this may have been many years after the event, so there is the possibility that some events have been forgotten or misremembered. Events that may be less traumatic may have been forgotten, and highly traumatic events may even have been mentally blocked out.

## Data Availability

ALSPAC data access is through a system of managed open access. The steps below highlight how to apply for access to the data included in this data note and all other ALSPAC data: Please read the ALSPAC access policy which describes the process of accessing the data and samples in detail, and outlines the costs associated with doing so. You may also find it useful to browse our fully searchable research proposal database which lists all research projects that have been approved since April 2011. Please submit your research proposal for consideration by the ALSPAC Executive Committee. You will receive a response within 10 working days to advise you whether your proposal has been approved.
