# Supplementary material for: Childhood life events of women enrolled in the Avon Longitudinal Study of Parents & Children (ALSPAC)
Source: Wellcome Open Res. 2023 Oct 18;8:294. Originally published 2023 Jul 10. [Version 2] doi: 10.12688/wellcomeopenres.19459.2 (PMC10457554; doi:10.12688/wellcomeopenres.19459.2)
Supplement: Peer review history

# Reviewer report: Reviewer response for version 1

**DOI:** 10.21956/wellcomeopenres.21556.r62704

**Published:** 2023-08-25

**Authors:** Berihun Assefa Dachew

**Affiliations:** 1 Curtin School of Population Health, Curtin University, Perth, Western Australia, Australia

The paper describes the variety of text data collected from women enrolled in the Avon Longitudinal Study of Parents & Children (ALSPAC) and explains the meticulous coding process used to analyze childhood life events.

 The article is well-written, has clear aims, and is easy to follow. Hence, I don’t have any major issues to comment on. However, to further benefit the readers, I recommend adding operational definitions on the severity of the trauma on page 12 of the article and/or as a footnote on tables 5a and b.

Are sufficient details of methods and materials provided to allow replication by others?

Yes

Is the rationale for creating the dataset(s) clearly described?

Yes

Are the datasets clearly presented in a useable and accessible format?

Yes

Are the protocols appropriate and is the work technically sound?

Yes

Reviewer Expertise:

Perinatal epidemiology, child adolescent mental health, and early life adversities.

I confirm that I have read this submission and believe that I have an appropriate level of expertise to confirm that it is of an acceptable scientific standard.

# Reviewer report: Reviewer response for version 1

**DOI:** 10.21956/wellcomeopenres.21556.r62700

**Published:** 2023-08-25

**Authors:** Alicia Matijasevich

**Affiliations:** 1 Department of Preventive Medicine, Faculty of Medicine FMUSP, Universidade de São Paulo, São Paulo, Brazil

Very interesting article, very well written, clear, and extremely useful for those working with epidemiological data in the field of trauma and adverse events throughout the life course. Congratulations to the authors for making ALSPAC's data available and explaining in detail the methodology of data collection and categorization of variables. This will facilitate harmonization with data from other studies.

 I have a few doubts regarding the manuscript:

 Page 6, first paragraph of the "Records" section: The following sentence is unclear: "The most commonly completed row was Year 0 (equivalent to age <1 year), which was completed by 11,563 respondents..." Does this refer to when the mother or the child was less than 1 year old? Or does it pertain to the previous year?

 Table 1: Does the row "1" indicate that 2.9% of individuals answered only one row? And does the row "17" indicate that 78.3% of individuals answered all 17 rows? It would seem more logical for most respondents to remember 1 or 2 episodes from childhood and not complete all 17 rows of the questionnaire.

 Table 2 shows a great diversity of countries/regions. This is interesting because I initially thought that the ALSPAC population would not have such a diverse range of origins, although the majority is from the UK.

 Does "zero" in Table 2c mean the same as "1" in Table 1?

 In Table 3a, I am unable to understand the meaning of the line "no one," and in Table 3c, the line "nobody."

 The complete list of events and their categorization provided in the article is very useful. It can serve as a foundation for researchers in the field to replicate the methodology and compare their results with those of ALSPAC. I was particularly struck by the relatively low frequency of severe traumatic events compared to the Brazilian cohorts I am more familiar with.

Are sufficient details of methods and materials provided to allow replication by others?

Yes

Is the rationale for creating the dataset(s) clearly described?

Yes

Are the datasets clearly presented in a useable and accessible format?

Yes

Are the protocols appropriate and is the work technically sound?

Yes

Reviewer Expertise:

Epidemiology

I confirm that I have read this submission and believe that I have an appropriate level of expertise to confirm that it is of an acceptable scientific standard.
